# Stereocomplexation of Poly(lactic acid) and Chemical Crosslinking of Ethylene Glycol Dimethacrylate (EGDMA) Double-Crosslinked Temperature/pH Dual Responsive Hydrogels

**DOI:** 10.3390/polym12102204

**Published:** 2020-09-25

**Authors:** Zhidan Wang, Jie Wu, Xiaoyu Shi, Fei Song, Wenli Gao, Shouxin Liu

**Affiliations:** Key Laboratory of Applied Surface and Colloid Chemistry, Ministry of Education, School of Chemistry and Chemical Engineering, Shaanxi Normal University, Xi’an 710119, China; 18189608643@163.com (Z.W.); 18375847342@163.com (J.W.); 18647847370@163.com (X.S.); 17563713257@163.com (F.S.); 17853465526@163.com (W.G.)

**Keywords:** double-crosslinked, polylactic acid, temperature/pH sensitive, stereo-complexation

## Abstract

Physical crosslinking and chemical crosslinking were used to further improve the mechanical properties and stability of the gel. A temperature/pH dual sensitive and double-crosslinked gel was prepared by the stereo-complex of HEMA-PLLA_20_ and HEMA-PDLA_20_ as a physical crosslinking agent, ethylene glycol dimethacrylate (EGDMA) as a chemical crosslinking agent, and azodiisobutyronitrile (AIBN) as an initiator for free radical polymerization. This paper focused on the performance comparison of chemical crosslinked gel, a physical crosslinked gel, and a dual crosslinked gel. The water absorption, temperature, and pH sensitivity of the three hydrogels were studied by a scanning electron microscope (SEM) and swelling performance research. We used a thermal analysis system (TGA) and dynamic viscoelastic spectrometer to study thermal properties and mechanical properties of these gels. Lastly, the in vitro drug release behavior of double-crosslinked hydrogel loaded with doxorubicin under different conditions was studied. The results show that the double-crosslinked and temperature/pH dual responsive hydrogels has great mechanical properties and good stability.

## 1. Introduction

Among the treatment approaches for many diseases, the oral route is one of the safest and promising ways to deliver drugs effectively [1,2]. As a drug carrier with good biocompatibility and strong practicability, the hydrogel has been widely applied in the field of medicine delivery in recent years [3,4]. Stimulus-responsive hydrogels (SRHs), also known as “smart” hydrogels, are being actively studied [5,6,7], and are generally defined as hydrogels that can change their swelling properties in response to external stimuli such as pH [8,9], light [10], electricity [11], magnetism [12], temperature [13,14], and redox [15]. The study of stimuli-responsive hydrogels has further improved the targeted release of drug-loaded hydrogels and expanded the application of hydrogels.

However, the poor mechanical properties of hydrogels have always been one of the obstacles hindering its development in the medical field [16,17]. In order to improve the mechanical properties of hydrogels, many new hydrogel materials have been widely studied such as the topological gel (TP gel) [18], dual-crosslinked hydrogels (DN gel) [19], and nanocomposite gels (NC gel) [20]. All improve the mechanical properties of the gel by changing the structure of the gel network. There are also many people who introduce functional particles into the gel network to improve the performance of the gel, and, at the same time, make the gel have the corresponding functions required by biomedical applications. In other words, these functional gels achieve the integration of structure and function. In order to achieve this goal, scientists have paid close attention to finding new cross-linking methods in recent years and found that hydrogels with better cross-linking can be obtained by adopting the method of double cross-linking [21,22,23,24]. Swelling performance is a complex property of gels [25,26], but most hydrogels have a high degree of swelling so that their high-water content and large pores usually lead to relatively rapid drug release, which can range from hours to days, and there are also problems in the convenience of the application [27].

During the treatment of diseases with hydrogels, it is necessary to develop various hydrogels that respond to the complex biological environment of the human body, and there are few reports on hydrogels that respond to dual stimuli of temperature and pH. As a biodegradable polymer material with good biocompatibility and non-polluting products, polylactic acid is widely used in the field of medicine [28,29]. In this article, polylactic acid with good biocompatibility is used as the substrate, and the stereo-complex formed by HEMA-PLLA_20_ and HEMA-PDLA_20_ (synthesized by the method of ring-opening polymerization with hydroxyethyl methacrylate (HEMA) as the initiator in the previous work [30,31]) was used as a physical crosslinking agent. Ethylene glycol dimethacrylate (EGDMA) was used as a chemical cross-linking agent [32,33,34] to synthesize physical-chemical double cross-linked hydrogel. The low critical solution temperature (LCST) of copolymer P(MEO_2_MA-*co*-OEGMA) can be adjusted by changing the feed ratio of 2-(2-methoxy ethoxy) ethyl methacrylate (MEO_2_MA) and oligo (ethylene oxide) methacrylate (OEGMA) [35,36,37]. The monomer diethylaminoethyl methacrylate (DEAEMA) makes the gel pH sensitive. The properties of the chemical cross-linked gel, physical cross-linked gel, and double cross-linked hydrogel as well as the in vitro drug release behavior of doxorubicin-loaded double-cross-linked hydrogels have been studied. By comparison, it was found that double cross-linked hydrogel has better mechanical properties and thermal stability and its swelling degree is significantly reduced when compared to physically crosslinked hydrogels.

## 2. Materials and Methods

### 2.1. Materials

L-lactide and D-lactide (99.0%) were purchased from Macleans (Shanghai, China). Hydroxyethyl methacrylate (HEMA, 99%) and ethylene glycol dimethacrylate (EGDMA, 98.0%) were purchased from J&k (Beijing, China), oligo(ethylene oxide) methacrylate (OEGMA, 95%, *M*_n_ = 475 g·mol^−1^), and 2-(2-methoxy ethoxy) ethyl methacrylate (MEO_2_MA, 95%, *M*_n_ = 188.22 g·mol^−1^) were obtained from TCI (Shanghai Development Co., Ltd., Shanghai, China). Diethylaminoethyl methacrylate (DEAEMA, 99%, *M*_n_ = 185.26 g·mol^−1^) was purchased from Alpha Aeso (Shanghai, China) and distilled under reduced pressure before use. 2,2-Azoisobutyronitrile (AIBN) was purified by recrystallization from methanol. The solvents used are all dried and double distilled water is used in aqueous solutions.

### 2.2. Synthesis

#### 2.2.1. Synthesis of HEMA-PDLA Macromonomer

The HEMA-PDLA macromonomer was synthesized by ring-opening polymerization (ROP) with hydroxyethyl methacrylate (HEMA) as the initiator and 1, 8-diazabicyclo [5.4.0] undec-7-ene (DBU) as the catalyst. A specific scheme has been detailed in previous work [31]. Hydroxyethyl methacrylate (HEMA) (0.121 mL, 1 mmol), D-lactide (1.73 g, 13 mmol), and dry CH_2_Cl_2_ (DCM) (50 mL) were added to a dry three-necked flask under dry nitrogen atmosphere. The mixture was stirred until all the monomers were dissolved and then DBU (1, 8-diazabicyclo [5.4.0] undec-7-ene) (20 μL, 0.13 mmol) was injected into the system. After stirring at room temperature overnight, the reaction was terminated after the addition of benzoic acid. The reaction mixture was concentrated and then precipitated twice in n-hexane. The polymers were vacuum dried at 35 °C. The HEMA-PLLA macromonomer is similar to the synthesis of HEMA-PDLA.

#### 2.2.2. Synthesis of Double-Crosslinked Dual Responsive Hydrogels

**Scheme 1:**
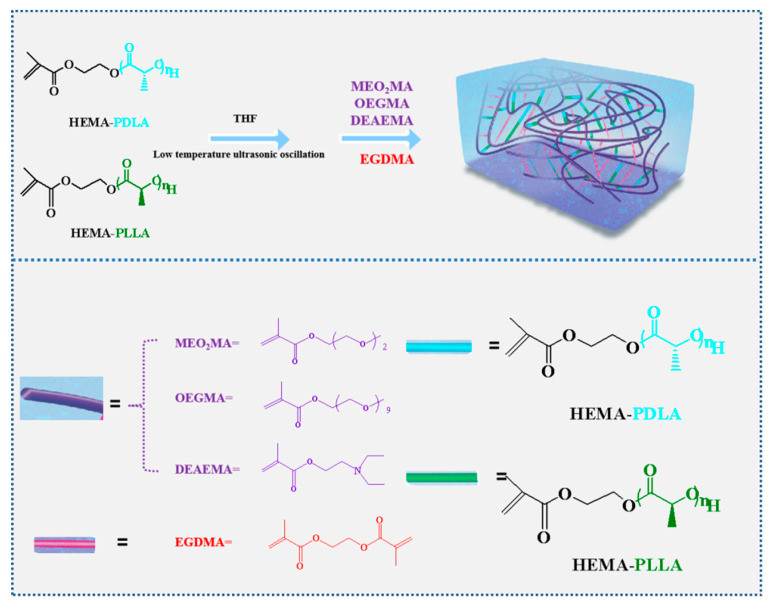
Synthesis of double-crosslinked temperature/pH dual responsive hydrogels.

The double crosslinked dual responsive hydrogel was prepared by free radical polymerization of the stereo-complex of a macromolecular monomer with a temperature-sensitive monomer MEO_2_MA and OEGMA, pH sensitive monomer DEAEMA, and chemical crosslinking agent EGDMA under the condition of AIBN as an initiator. Synthesis of a specific process is as follows. The macromonomer HEMA-PLLA_20_ (0.1 g, 0.01 mmol) and HEMA-PDLA_20_ (0.1 g, 0.01 mmol) are dissolved in 2 mL of tetrahydrofuran (THF), followed by low-temperature ultrasound for 30 min for full complexation, and then the solvent was evaporated under reduced pressure. The monomers MEO_2_MA (0.416 mL, 2.39 mmol), OEGMA (0.052 mL, 0.126 mmol), DEAEMA (0.505 mL, 2.52 mmol), and EGDMA (0.005 mL, 0.01 mmol) were added to the dry and clean round bottom flask and homogenized by stirring under the protection of nitrogen for 30 min. Then the stereo-complex of the macromonomer and AIBN (0.005 g, 0.030 mmol) are added to the reaction mixture and stirred until the solution is uniform. The reaction system was sealed and placed in a 70 °C oil bath, and the reaction was stirred for one hour under nitrogen. The synthesis steps are shown in Scheme 1.

#### 2.2.3. Synthesis of Chemical Crosslinked Gel

Using ethylene glycol dimethacrylate as a chemical crosslinking agent, the macromolecular monomer HEMA-PLLA were polymerized with MEO_2_MA, OEGMA, DEAEMA, and EGDMA on free radical polymerization to prepare the chemical crosslinked gel. The synthesis process is as follows. The monomers MEO_2_MA (0.416 mL, 2.39 mmol), OEGMA (0.052 mL, 0.126 mmol), DEAEMA (0.505 mL, 2.52 mmol), EGDMA (0.005 mL, 0.01 mmol), macromolecular monomer HEMA-PLLA_20_ (0.1 g, 0.01 mmol), and AIBN (0.005 g, 0.030 mmol) were added to the dry and clean round bottom flask, and the solution was homogenized by stirring under the protection of nitrogen for 30 min. The reaction system was sealed and placed in an oil bath at 70 °C and stirred with nitrogen for one hour.

### 2.3. Fourier-Transform Infrared Spectroscopy (FTIR)

Fourier transform infrared (FTIR) was measured by a Bruker Fourier-transform infrared spectroscopy (FTIR) (Tensor 27, Bruker Corporation, Karlsruhe, Germany). Specifically, the gel sample fully swollen in absolute ethanol is mixed with KBr, pre-dried to make it uniformly mixed, then dried in a vacuum-drying oven at 70 °C for 12 h, and pressed at room temperature before measurement.

### 2.4. The Wide-Angle X-ray Diffraction (WAXD) Analysis of the Hydrogel

The wide-angle X-ray diffraction (WAXD) analysis was performed on Ni-filtered Cu Kα (λ = 0.154 nm) at 25 °C on the D8 Advance diffractometer from Bruker, Karlsruhe, Germany, with a 2*θ* scanning rate of 2°/min and a scanning range of 5−40°.

### 2.5. Swelling and Deswelling Properties of the Hydrogel

The determination of the swelling kinetics of the gel is carried out by weighing the gel swelled under different conditions. Specifically, the fully dried gel samples were placed in the secondary water of (1) pH = 7, 25 °C, (2) pH = 5, 25 °C weighed after a fixed time interval (filter paper was used to absorb the surface moisture of the gel before each weighing), weighed three times and recorded the data, and the swelling ratio was calculated, according to the following formula.

$$\left[\mathrm{Swelling}\;\mathrm{ratio}\right]=\left(W_{t}-W_{d}\right)/W_{d}$$

where *W*_d_ is the mass of dry gel (g) and *W_t_* is the mass of the gel taken out at a fixed time point during the swelling process (g).

The de-swelling kinetics of the gel is determined by immersing the fully swollen hydrogel in double distilled water at 40 °C or pH = 9 (the operation method is the same as above). We calculated its water retention capacity according to the following formula.

$$\left[\mathrm{Water}\;\mathrm{retention}\right]=\left(W_{t}-W_{d}\right)/W_{s}$$

where *W*_d_ is the dried gel weight, *W*_s_ is the weight of the gel swelling equilibrium, and *W_t_* is the weight of the gel at a given time during de-swelling.

### 2.6. The Scanning Electron Microscope (SEM) Analysis of the Hydrogel

TM 3030 desktop scanning electron microscope (SEM) was used to observe the microscopic morphology of the gel in a low vacuum mode. The gel with swelling equilibrium under different conditions was quickly frozen with liquid nitrogen to better retain the gel morphology. Then the gel was dried by a freeze dryer. Before observation, 80 s of gold spray treatment is needed to improve the conductivity of the sample.

### 2.7. Thermal Analysis of the Hydrogel

Using a thermal analysis system (TGA) (TGA Q600SDT, TA Instruments, Milford, MA, USA) in a nitrogen atmosphere with a temperature range of 25~600 °C and a heating rate of 10 °C/min to measure the thermal properties of the gel. The thermogravimetric data and the first weight loss temperature should be recorded to compare the thermal stability of the gel.

### 2.8. Mechanical Properties Analysis of the Hydrogel 

The mechanical properties of the gel were measured by DMA Q800 dynamic viscoelastic spectrometer. Five newly prepared double cross-linked gels, chemical gels, and physical gels were used to intercept disc-shaped gel samples with a puncher. DMA Q800 dynamic viscoelastic spectrometer was used to test the storage modulus of the hydrogel in a static compression multi-frequency strain mode, and the mechanical strength was described by comparing the storage modulus of the gel.

### 2.9. Drug Sustained Release Analysis of the Hydrogel 

The drug release research of the gel is mainly carried out by the ultraviolet rays (UV) analysis method.

Preparation of the drug-loaded gel: Put 5 mg of the dried hydrogel into 1 mg/mL doxorubicin solution (5 mg doxorubicin, 5 mL THF), swell at room temperature for 72 h, and take it out and washed with THF to remove doxorubicin on the surface of the gel, then placed at room temperature for 2 d, and lastly dried in a vacuum oven at 40 °C for 4 d.

Determination of the maximum absorption wavelength: Doxorubicin is used as a hydrophobic drug model to study the in vitro release of drugs from the gel. Doxorubicin was dissolved in a phosphate buffered saline solution (PBS) buffer solution with pH = 7.4. A blank PBS solution was used as a control group. A UV spectrophotometer was used to scan the spectrum in the range of 200–600 nm to determine that the maximum absorption wavelength of doxorubicin was 233 nm.

Determination of the standard curve: PBS buffer solution is used as the solvent, and the prepared concentrations are 1 μg·mL^−1^, 2 μg·mL^−1^, 5 μg·mL^−1^, 10 μg·mL^−1^, 15 μg·mL^−1^, doxorubicin solutions of 20 μg·mL^−1^, 25 μg·mL^−1^, and 50 μg·mL^−1^, and the absorbance values of the above concentrations were measured at the maximum absorption wavelength of 233 nm. Then a standard curve with absorbance as the ordinate and concentration as the abscissa was fitted, and the linear regression equation obtained was the following equation.

Abs = 0.05116*c* + 0.00519, *R*^2^*=* 0.99833

Among them, Abs represents the absorption intensity of doxorubicin at 233 nm, and *c* represents the release concentration of doxorubicin.

Cumulative drug release measurement: 0.05 g of hydrogel containing doxorubicin (0.1 wt %) was put into 150 mL of PBS (pH = 7.4) buffer solution at 25 °C, 37 °C, and 45 °C, and taken out at regular intervals of 4 mL of buffer solution. Its absorbance was measured at the maximum wavelength, and then the same amount of fresh PBS buffer solution was added in the drug release system. Then the drug release was obtained at different times according to the standard curve. Lastly, the cumulative release rate of doxorubicin was calculated according to the following formula.

$$\mathrm{Cumulative}\;\mathrm{release}\;\left(\%\right)=\frac{V_{e}\sum_{1}^{n-1}c_{i}+V_{0}c_{n}}{m_{\mathrm{drug}}}\times 100.$$

Among them, *V*_e_ is the volume of the solution taken from the PBS buffer solution each time, the *V*_0_ is the total volume of the solution that began to be released, the *c*_i_ is the concentration of the drug released from the micelles when the solution was removed for the *i* time, and *n* is the number of times the solution is taken out. The *m*_drug_ is the total mass of doxorubicin for drug release.

## 3. Results and Discussion

### 3.1. Synthesis and Characterization of Double-Crosslinked Dual Responsive Hydrogels

The poly (lactic acid)-based double crosslinked hydrogel was synthesized by the stereo-complex microcrystal formed between PLLA and PDLA as the physical crosslinking and agent EGDMA as chemical crosslinking. We used the macromonomer with a degree of polymerization of 20 synthesized in the previous work to pre-mix it to form an active PLA complex, and then mixed it with the temperature-sensitive monomer MEO_2_MA and OEGMA, pH-sensitive monomer DEAEMA, and chemical crosslinker EGDMA. AIBN was used as an initiator to react for 1 h at 70 °C while a double cross-linked double stimulus responsive hydrogel was synthesized. Compared with physical crosslinked hydrogels [30], double crosslinked hydrogels have better mechanical strength and properties. We synthesized the hydrogels with the chemical crosslinking agent EGDMA as a variable for comparison. The specific feeding materials are shown in Table 1.

**Figure 1:**
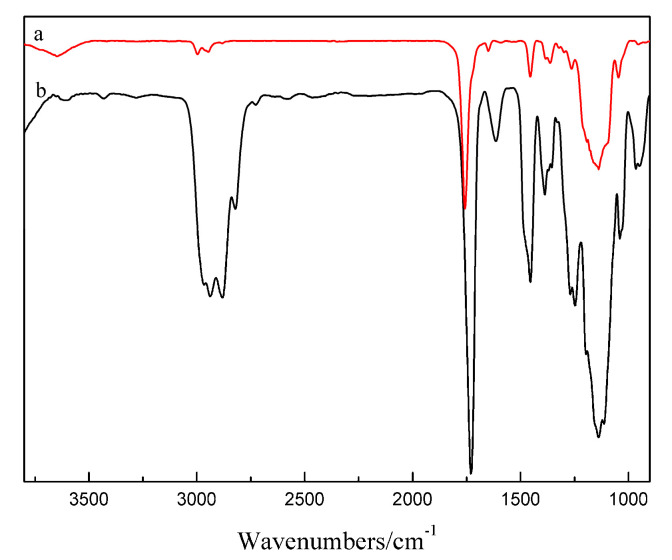
Fourier-transform infrared spectroscopy (FTIR) spectra of (**a**) chemical cross-linked gel5, (**b**) the double-crosslinked gel.

Figure 1a,b shows the FTIR spectrum of the chemical cross-linked gel and the double-crosslinked gel, respectively. It can be seen that the peak of the two gels at 1254 cm^−1^ is C–O–C stretching vibration, 1469 cm^−1^ and 2933 cm^−1^ are the bending and stretching vibration peaks of CH, and the peak at 1133 cm^−1^ is the vibration absorption peak of –N^+^(CH_2_)_3_–. These characteristic absorption peaks coincide through chemical cross-linking condensation. The characteristic peak of C=O in gel appears at 1760 cm^−1^ (Figure 1a), while the characteristic peak of C=O in a double-crosslinked gel appears at 1736 cm^−1^ (Figure 1b). The absorption peak shifted, which indicates that the physical crosslinking of PLLA and PDLA occurred in the double-crosslinked gel. The appearance of these peaks proved that the synthesis of the double-crosslinked hydrogel was successful.

### 3.2. The Wide-Angle X-ray Diffraction (WAXD) Analysis of the Hydrogel

**Figure 2:**
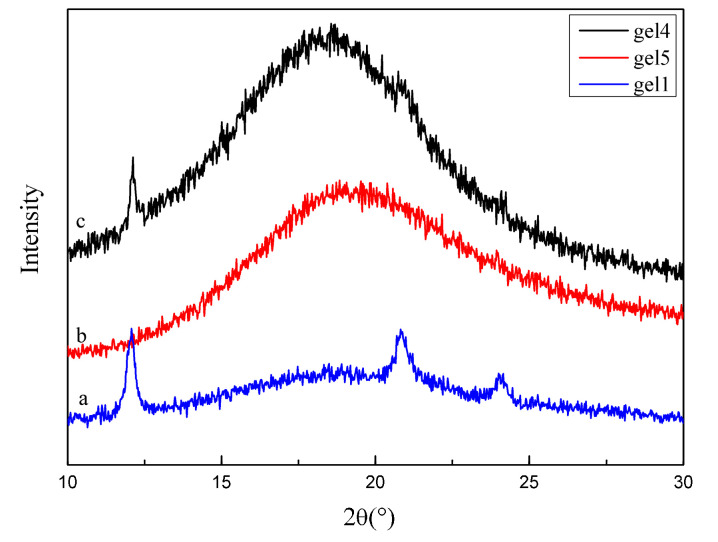
The wide-angle X-ray diffraction profile for (**a**) physical crosslinked hydrogels, (**b**) chemical crosslinked hydrogels, and (**c**) double-crosslinked hydrogels.

The stereo-complex microcrystals of PLA can be verified by wide-angle X-ray diffraction. Figure 2a–c shows the WAXD curves of a physical cross-linked hydrogel, a chemical cross-linked hydrogel, and a double cross-linked hydrogel, respectively. As the figure shows, no clear diffraction peak appears in the chemical cross-linked hydrogel, and the curve tends to be flat. However, clear crystallization peaks appear in both the physical cross-linked hydrogel and the double cross-linked hydrogel at 2θ = 12°, the diffraction peak of the double-crosslinked hydrogel at 2θ = 21°, 24° is not clear, which may be because there are two ways of cross-linking in the double-cross-linked gel. This results in incomplete physical crosslinking, so the diffraction peak is not clear. According to the literature, the diffraction peak at 2θ = 12° can prove that the PLLA/PDLA stereo-complex physical crosslinking has occurred in the double crosslinked gel [38].

### 3.3. Temperature and pH Sensitivity of the Hydrogel

**Figure 3:**
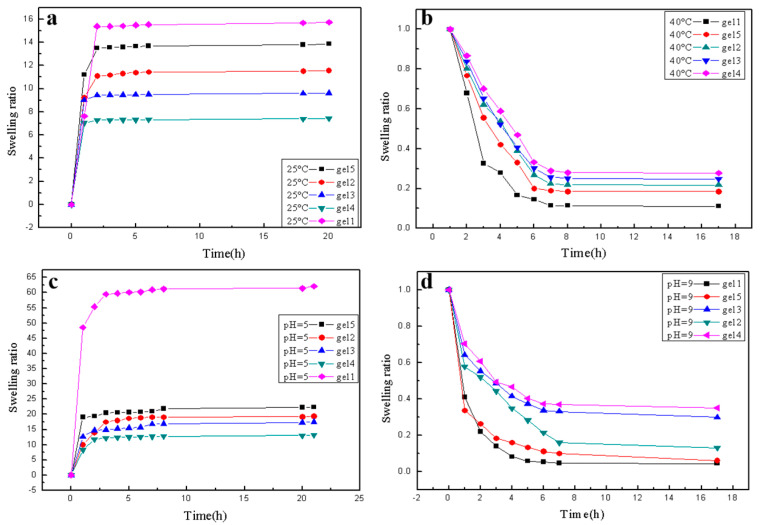
Swelling ratios of hydrogels (**a**) at 25 °C, pH = 7. (**b**) at 40 °C pH = 7. (**c**) at 25 °C, pH = 5, (**d**) at 25 °C, pH = 9.

In order to verify the temperature sensitivity and pH sensitivity of double-crosslinked hydrogels, we used a physical cross-linked gel, a chemical cross-linked gel, and a double cross-linked gel to conduct swelling kinetic experiments under different conditions. Figure 3a is the swelling kinetic curves of the physical cross-linked gel, the chemical cross-linked gel, and the double cross-linked gel at a temperature of 25 °C and pH = 7. The figure shows that the gels of different cross-linking methods all show clear water swelling. This is because the hydrophilic part of the gel P(MEO_2_MA-*co*-OEGMA) binds to water molecules at 25 °C to form hydrogen bonds. The swelling rate of hydrogels was faster in the first two hours and leveled off after two hours. Different cross-linked hydrogels had different swelling rates. Among them, the physical cross-linked hydrogel has the highest swelling rate at about 15.22, which is followed by the chemical cross-linked hydrogel.

The swelling rate is about 13.89. This is because gel5 is forced by the covalent bond and gel1 is forced by a hydrogen bond. As we all know, the covalent bond force is stronger than the hydrogen bond force, which causes the softness and elasticity of the inner wall of the three-dimensional network in gel5 being less than that of gel1. Therefore, the internal hydrophilic segment of gel1 is easier to form hydrogen bonds with water molecules. The swelling rate of double-crosslinked hydrogels was low, and the swelling rate gradually decreased with the increase of the chemical crosslinking dose. When the chemical crosslinking agent content was 7%, the lowest was about 7.12. This is because double-crosslinked hydrogels have both chemical and physical crosslinks, and there are more crosslinking points than single crosslinks. Moreover, with the increase of chemical cross-linking agents, the number of crosslinks in the gel network increases, which leads to a small pore size of the gel. Thus, the swelling rate of double-crosslinked hydrogels is low. This means that the water absorption capacity of double-crosslinked hydrogels is not strong. Therefore, they will not cause local edema in the affected area due to hydrogel administration in practical therapeutic applications. This feature is of great significance for the drug release of hydrogels in a human environment.

To further verify the temperature sensitivity of the double-crosslinked hydrogel, we transferred the hydrogel fully swelled in the secondary water at 25 °C and pH = 7 to 40 °C and pH = 7. Figure 3b shows the hydrogel deswelling kinetic curve of the gel. The gel has a tendency to shrink and lose water at 40 °C, which proves that the gel has temperature sensitivity. This is because, when the temperature rises to 40 °C, the hydrogen bond breaks, and the temperature-sensitive polymer chain P(MEO_2_MA-*co*-OEGMA) chain begins to shrink [39] by squeezing water out of the gel. Thus, this shows a state of de-swelling. Synthesizing the swelling kinetics and de-swelling kinetics curves of the hydrogel proves that the double crosslinked hydrogel has temperature sensitivity.

We conducted swelling kinetic experiments on the three gels at a temperature of 25 °C and pH = 5 to verify their pH sensitivity. As shown in Figure 3c, the gels of different cross-linking methods all exhibit clear water swelling, and the swelling rate of the gel increases with time. This is because, at pH = 5, the DEAEMA unit changes from unprotonated to fully protonated with the increase of time, and there is electrostatic repulsion in the gel, which promotes the molecules in the gel to present the stretching state. This leads to the loose structure of the gel network and the increase of the swelling rate. It can be seen from the figure that the swelling rate of double-crosslinked hydrogels was low, and decreased gradually with the increase of a chemical crosslinking dose. The lowest swelling rate was 12.36 when the chemical crosslinking agent content was 7%. This is because double-crosslinked hydrogels have more crosslinking points. Moreover, with the increase of chemical crosslinking agents, the number of crosslinking points in the gel network increases, which leads to a small pore size of the gel. Therefore, the swelling rate of double-crosslinked hydrogels is low. The de-swelling kinetic curve shown in Figure 3d further validates the pH sensitivity of the double cross-linked hydrogel. It can be seen that several hydrogels have a tendency to shrink with time, and tend to shrink after 7 h. This is because deprotonation occurs in the DEAEMA unit at pH = 9, which makes the gel uncharged. Hydrogen bonds form in the gel to play a major role, which results in a tighter network structure of the gel and a shrinking state of the gel. Therefore, this shows a de-swelling state.

### 3.4. Thermal Analysis of the Hydrogel

**Figure 4:**
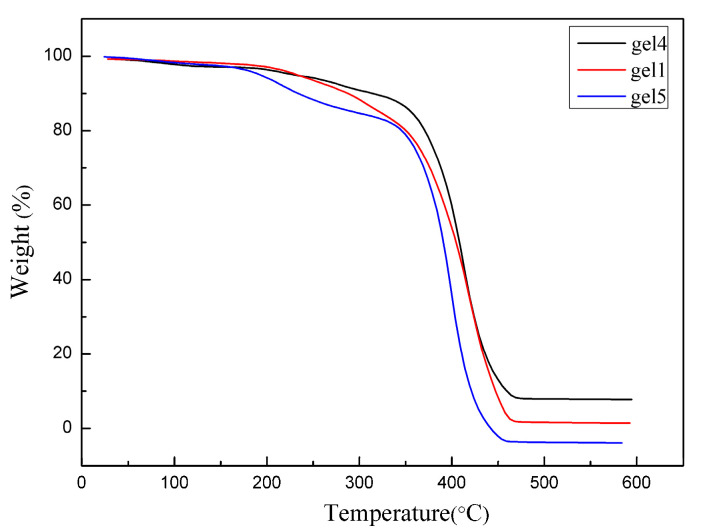
Thermogravimetric analysis curves of physically-crosslinked hydrogels and chemically-crosslinked hydrogels and double-crosslinked hydrogels.

The thermal stability of three gels was studied by thermogravimetric analysis (TGA). According to literature reports, the thermal degradation temperature refers to the temperature at the first weight loss. Figure 4 shows the TGA curves of three hydrogels. It can be seen from the figure that chemical cross-linked hydrogels lose weight twice at about 190 and 320 °C, which indicates that chemical cross-linked hydrogels have poor thermal stability. However, both the physical cross-linked hydrogel and the double cross-linked hydrogel showed only one weight loss at about 300 °C and 340 °C, which indicates that the two gels have good thermal stability. This is judged by the temperature of the first thermal degradation. The thermal degradation temperature of the double cross-linked gel is higher than that of the physical cross-linked hydrogel. These results indicate that the double cross-linked hydrogels have good thermal stability.

### 3.5. Scanning Electron Microscope (SEM) Analysis of the Hydrogel

**Figure 5:**
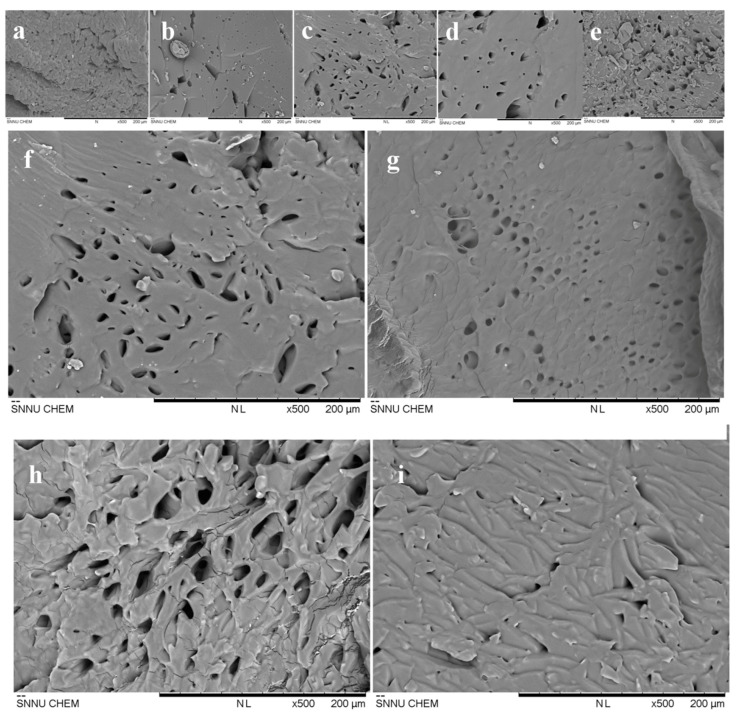
Morphology of freeze-dried gels at pH = 7, 25 °C (**a**) gel4, (**b**) gel3, (**c**) gel2, (**d**) gel5, and (**e**) gel1. Morphology of freeze-dried gel2 (**f**) at pH = 7, 25 °C, (**g**) pH = 7, 40 °C, (**h**) pH = 5, 25 °C, and (**i**) pH = 9, 25 °C.

The three-dimensional network structure of the gel can be observed directly by using a tabletop scanning electron microscope (SEM). The sample preparation is to preserve the network structure inside the gel by freeze-drying the hydrogel fully swollen under different conditions in the liquid nitrogen environment. The size of the three-dimensional network of the gel can be judged by the size of the pore size, and the change of the gel in different environments. Figure 5a–e shows SEM images of freeze-dried gels 4, 3, 2, 5, 1 at pH = 7 and 25 °C, respectively. From the figure, we can see that they have a clear three-dimensional network structure in which the chemical cross-linked hydrogel (gel5) has the largest pore size, which is followed by the physical cross-linked hydrogel (gel1). The pore size of the double cross-linked hydrogel is small, and, as the content of the chemical cross-linking agent increases, the pore size gradually decreases. When the content of the chemical cross-linking agent is 7%, the pore size is the smallest. This is because a double-cross-linked hydrogel has two kinds of cross-linked structures. When the physical cross-linking points are the same, the more chemical cross-linking agents there are, the more crosslinking points there are, and the tighter the cross-linking is. The gel formed has a tight three-dimensional network structure, which means gel4 has the smallest pore size.

Previous studies on the swelling kinetics and de-swelling kinetics of gels have demonstrated that the synthesized double cross-linked gels have temperature and pH sensitivity. Observing the pore size changes in the network structure of the gel under different conditions through SEM can also verify the temperature and pH sensitivity of the gel. Figure 5 shows scanned photos of freeze-dried gel2 at pH = 7 and 25 °C (Figure 5f), pH = 7 and 40 °C (Figure 5g), pH = 5 and 25 °C (Figure 5h), pH = 9 and 25 °C (Figure 5i). It can be seen from the figure that changes in temperature and pH have a great influence on the network state of the gel. By comparing SEM images in Figure 5f,g, it is found that the temperature has a certain effect on the three-dimensional network structure of the double-crosslinked hydrogels. At pH = 7 and 25 °C, the gel is in a state of swelling with water absorption, and the pore size of the gel structure is large. However, at pH = 7 and 40 °C, the gel is in a state of shrinkage and dehydration, and the pore size is small. The reason is that, at higher temperatures, the hydrogen bond formed by the hydrophilic part P(MEO_2_MA-co-OEGMA) and water molecules is broken, and the temperature-sensitive polymer chain starts to shrink, which reduces the pore size of the gel and further indicates that the double crosslinked hydrogel is temperature-sensitive. Figure 5h,i shows that pH also has a great influence on the network structure of the hydrogel. At pH = 5 and 25 °C, the gel has a loose network structure and a large pore size. At pH = 9 and 25 °C, the gel has a compact structure and the pore size becomes significantly smaller. The reason is that, at higher pH values (pH > pKa), the DEAEMA unit is deprotonated, the hydrogen bond is formed by the hydrophilic part P(MEO_2_MA-co-OEGMA), water molecules play a major role, and the polymer chain PDEAEMA shrinks, which results in a tighter network structure of the gel. It further illustrates that the double crosslinked hydrogel has pH sensitivity.

**Figure 6:**
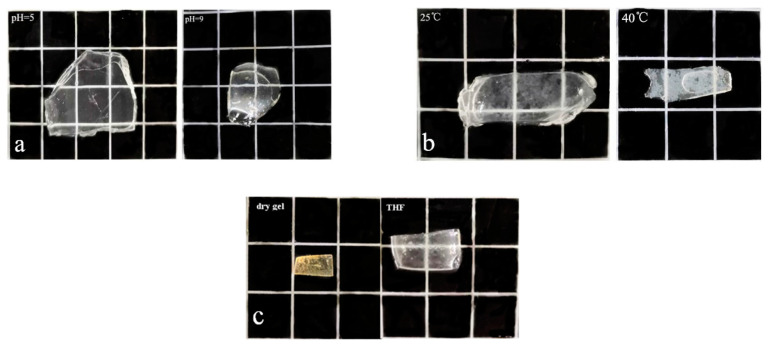
Photographs of gel2 (**a**) 25 °C, pH = 5 and pH = 9, (**b**) pH = 7, 25 °C and 40 °C, (**c**) 25 °C dry gel and swelled gel in tetrahydrofuran (THF).

Figure 6 is a digital photo of gel2 in different environments. The side length of each small grid in the picture is one centimeter. Figure 6a shows the swelling photos of gel2 at the temperature of 25 °C and pH values of 5 and 9, respectively. By comparison, it can be seen that gel2 is fully swollen at pH = 5 and shrank at pH = 9, which indicates that the double cross-linked hydrogel has pH sensitivity. Figure 6b is the swelling photo of the gel at pH 7 and temperatures of 25 °C and 40 °C, respectively. It can be seen by comparison that gel2 swells sufficiently at 25 °C and shrinks at 40 °C, which indicates that the double-crosslinked hydrogel has temperature sensitivity. Figure 6c is a comparison diagram of the gel before and after swelling in THF at room temperature. The gel has swelled in THF, which further shows that the synthesized double-crosslinked hydrogel is amphiphilic.

### 3.6. The Mechanical Properties of Hydrogels

**Figure 7:**
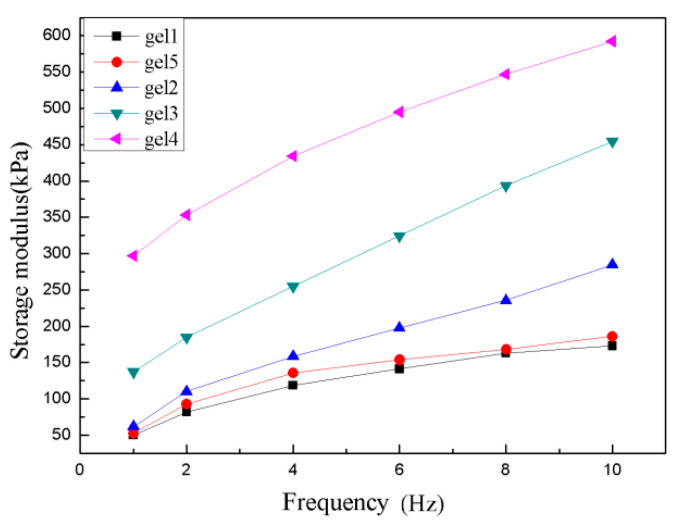
Variation curve of storage modulus with frequency of hydrogels.

The storage modulus of the hydrogel can reflect its physical and mechanical properties very well. On this basis, we studied the variation curve of the storage modulus of the hydrogel with different cross-linking methods with frequency. Figure 7 shows the changes of the storage modulus of gel1, 2, 3, 4, and 5 with frequency, respectively. It can be seen that, when the frequency is 10 Hz, the storage moduli of the gel are 170 kPa, 283 kPa, 455 kPa, 590 kPa, and 185 kPa, respectively, which indicates that the five hydrogels have good mechanical strength. However, it can be clearly seen from the figure that the storage modulus of the double-crosslinked hydrogel is relatively high and varies with the content of the chemical cross-linking agent. This is because, in terms of the stereo-complex of PLA at the same DP (degree of polymerization), the physical crosslinking point of the gel is the same, and the internal network structure of the gel varies with the content of a chemical crosslinking agent. Gel4 has the highest content of chemical cross-linking agents when forming a gel. The most cross-linking points result in a tight three-dimensional network structure and small pore size. Therefore, the gel has the best mechanical strength. As we all know, the swelling ratio of hydrogels is inversely proportional to the gel strength. Therefore, the comparison results of the gel strength of gel1, 2, 3, 4, and 5 are consistent with their swelling ratio comparison results.

### 3.7. Drug Sustained Release of the Hydrogel

**Figure 8:**
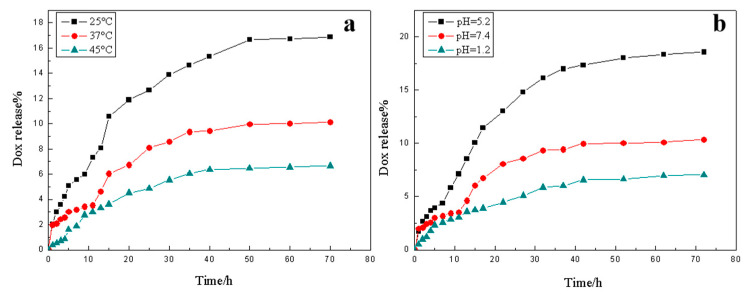
Drug release curve of the dox-loaded hydrogel in phosphate buffered saline solution (PBS) with (**a**) pH = 7.4 at a different temperature, (**b**) 25 °C at a different pH.

Doxorubicin (DOX) is a typical chemotherapy drug that can be used to treat neuroblastoma, bladder cancer, thyroid cancer, etc. However, it has been found in practical applications that it may cause multiple organ toxicity in patients [40]. Therefore, it is very necessary to choose an effective medical carrier for loading. Therefore, we selected the hydrophobic drug doxorubicin gel4 as a drug carrier for drug release studies. We conducted a sustained-release study of gel4 under physiological conditions (PBS, pH = 7.4) at 25 °C, 37 °C, and 45 °C, respectively. Figure 8a is the release curve of gel4 loaded with doxorubicin in phosphate buffer solution of pH = 7.4 at different temperatures. It can be seen from the figure that the drug-loaded gel releases doxorubicin slowly. When the drug-loaded gel4 is at 25 °C, the cumulative release ratio of doxorubicin in 3 days is 17%. This is because doxorubicin is a hydrophobic drug that mainly accumulates in the hydrophobic chain region and does not diffuse well in water. With the temperature increases, the drug release ratio of the drug-loaded gel decreases. At 45 °C, the release ratio of doxorubicin in three days is only 5.7%. The main reason is that, when the temperature is higher than the abrupt transition temperature of the gel, the hydrophilic polymer P(MEO_2_MA-*co*-OEGMA) self-aggregates and the gel is in the state of de-swelling. This forms a hydrophobic network structure, which results in the enhanced interaction between hydrophobic drugs and the gel. The hydrophobic drugs are difficult to release from the gel.

Figure 8b was the drug release curve of the gel4 loaded with doxorubicin in phosphate buffer solutions of different pH. It can be seen from the figure that, when the drug-loaded gel4 is pH = 5.2, the cumulative release ratio of doxorubicin in three days is 18%. This is because, when the pH of the solution is lower than the p*K*a value of the ionizable amino group, PDEAEMA in the network is protonated. Electrostatic repulsion occurs in the network chain, which results in the spatial separation of the polymer chain by loosening the network structure of the gel, and promoting the release of doxorubicin in water. At pH = 7.4 and pH = 1.2, the cumulative release ratio of doxorubicin in three days is less than 10%, which indicates that the drug-loaded gel has less drug leakage in normal tissues, and, thereby, reduces toxicity to normal tissues.

The temperature and pH of the two most important parameters in human and biological systems have great influence on the drug release of the gel. Moreover, the research shows that, compared with the physical cross-linked gel, the double-crosslinked gel not only reduces the swelling degree and enhances the mechanical strength, but also has no significant influence on the controlled release of hydrophobic drugs. The total release ratio of the three-day drug is only 3% less, which indicates that the double-crosslinked gel has greater application potential in the drug delivery system.

## 4. Conclusions

In this article, a novel double-crosslinked amphiphilic network gel was formed by the physical crosslinking of the macromolecular monomers HEMA-PLLA_20_, HEMA-PDLA_20_, and chemical crosslinking agent EGDMA. The temperature sensitive monomers MEO_2_MA, OEGMA, and pH sensitive monomer DEAEMA were polymerized to the macromolecular monomers by free radical polymerization, which makes the hydrogel temperature and pH desensitized and amphiphilic. Through a series of characterization work, the double-crosslinked and temperature/pH dual responsive hydrogels synthesized by the combination of physical crosslinking and chemical crosslinking has good biocompatibility, great mechanical properties, and stability. It can provide a carrier for drugs to achieve a slow-release effect and will have a greater application prospect in drug delivery systems.

## Figures and Tables

**Table 1:** Synthesis data of hydrogels.

Sample	*n*(HEMA-PLLA_20_) (mol)	*n*(HEMA-PDLA_20_) (mol)	[DEAEMA]/[(OEGMA+MEO_2_MA)] (*n*:*n*)	EGDMA (%)
gel1	0.08	0.08	0.3	0
gel2	0.08	0.08	0.3	3
gel3	0.08	0.08	0.3	5
gel4	0.08	0.08	0.3	7
gel5	0.16	0	0.3	100

DEAEMA: diethylaminoethyl methacrylate, MEO_2_MA: 2-(2-methoxy ethoxy) ethyl methacrylate, OEGMA: oligo(ethylene oxide) methacrylate, EGDMA: ethylene glycol dimethacrylate. The ratio between MEO_2_MA and OEGMA is 95:5.
